# Myocardial fibrosis in congenital heart disease

**DOI:** 10.3389/fped.2022.965204

**Published:** 2022-11-18

**Authors:** Blanca Gordon, Víctor González-Fernández, Laura Dos-Subirà

**Affiliations:** Integrated Adult Congenital Heart Disease Unit, Vall d’Hebron University Hospital—Santa Creu i Sant Pau University Hospital, Barcelona, Spain

**Keywords:** myocardial fibrosis, fibrosis, congenital heart disease, cardiac magnetic resonance (CMR), collagen biomarkers, tetralogy of Fallot, systemic right ventricle, myocardial interstitial fibrosis

## Abstract

Myocardial fibrosis resulting from the excessive deposition of collagen fibers through the myocardium is a common histopathologic finding in a wide range of cardiovascular diseases, including congenital anomalies. Interstitial fibrosis has been identified as a major cause of myocardial dysfunction since it distorts the normal architecture of the myocardium and impairs the biological function and properties of the interstitium. This review summarizes current knowledge on the mechanisms and detrimental consequences of myocardial fibrosis in heart failure and arrhythmias, discusses the usefulness of available imaging techniques and circulating biomarkers to assess this entity and reviews the current body of evidence regarding myocardial fibrosis in the different subsets of congenital heart diseases with implications in research and treatment.

## Introduction

The history of congenital heart disease (CHD) is a history of hope and success. Since the first ligation of a patent ductus arteriosus accomplished by Gross and Hubbard on August 8, 1938 (1), an unstoppable flow of innovative and creative surgical and interventional techniques has dealt with the most unimaginable cardiac malformations. Thousands of children destined to die soon after birth have been able to reach adulthood and, in most cases, develop a near-normal life. But the history of CHD is also paved with uncertainties. These repaired hearts are frequently afflicted with long-term complications, predominantly heart failure (HF) and arrhythmias. Our understanding of the mechanisms behind these complications and the available treatment options is based on the knowledge borrowed from acquired heart diseases and raises more questions than answers. The singularities of CHD should compel us to look beyond the ordinary and seek personalized, precision medicine for our patients.

During the last years, the myocardial interstitium (MI) has gone from the role of innocent bystander to a preeminent position in the pathogenesis of long-term cardiovascular complications, mainly HF. The MI is not a passive entity, but rather a complex and dynamic microenvironment that undergoes constant turnover: it ensures structural myocardial integrity, executes the repair response after injury, provides the means for force transmission throughout the cardiac cycle, translates myocyte shortening into overall ventricular pump function and facilitates communication between cells (2).

The accumulation of fibrillar collagen in the interstitial space, the so-called myocardial interstitial fibrosis, has been identified as a major cause of myocardial dysfunction. It not only distorts this normal architecture but also impairs its biological function and properties.

The term “interstitial myocardial disease” was first coined in 1989 by Weber (3) in reference to maladaptive effects on interstitial remodeling in response to altered hemodynamic loading conditions and/or myocardial damage. These changes, including fibroblast activation, formation of myofibroblasts, inflammation and altered expression of metalloproteinases, ultimately lead to myocardial fibrosis (MF) (4).

Altered hemodynamic conditions are a common feature of most CHDs, both before and after palliation or complete repair. Therefore, MF is expected to be one of the main maladaptative mechanisms behind the progressive deterioration of ventricular function in our patients and a potential therapeutic target.

This paper aims to summarize current knowledge about MF in terms of pathophysiology, diagnostic tools and potential treatments, focusing on the CHD population.

## Histopathology, mechanisms and types of myocardial fibrosis

For a simplified description, the myocardium can be dichotomized into two components. The cellular component is mainly represented by cardiomyocytes, occupying 75% of the myocardium but only conforming one-third of the cell population. The rest is constituted of endothelial cells, vascular smooth muscle cells, fibroblasts, macrophages and mast cells.. The non-cellular component of the myocardium is also located in the interstitial space and mainly comprises fibrillar collagen (95% of the extracellular matrix) but has also other constituents: proteoglycans, glycosaminoglycans and different bioactive signaling molecules (2, 5).

Among the cellular constituents, fibroblasts are responsible for the synthesis and maintenance of cardiac connective tissue. Fibroblasts can migrate to areas of damaged myocardium (i.e., surgical injury or myocardial infarction) where they trans-differentiate into myofibroblasts, which are crucial cells for cardiac remodeling and fibrosis (6). A myriad of chemokines, cytokines and growth factors are secreted in the injured myocardium and stimulate the activation and differentiation of fibroblasts. These bioactive elements are produced in response to different stimuli, including mechanical stress (7). The healing process includes degradation of the damaged tissue and subsequent production, cross-linking and maturation of a new extracellular matrix (ECM). Although myofibroblasts are the major source of ECM, other cells, like monocytes, macrophages, cardiomyocytes and non-differentiated fibroblasts contribute to the process by secreting pro-fibrotic growth factors (8).

As for non-cellular components, fibrillar collagen is the cornerstone, with five isoforms in the myocardial interstitium: collagen type I, III, IV, V and VI. Type I collagen is the predominant, representing 80% of the total. It offers the greatest tensile strength (comparable to steel) and represents the major determinant of myocardial stiffness. Type III collagen has more elastic characteristics, representing 10% of collagens, and the other types(IV, V and VI) constitute the remaining 10% (9, 10).

From a histopathological point of view, two types of MF should be distinguished. The *reparative*, replacement or focal myocardial fibrosis (FMF) develops as a healing mechanism at the site of a previous myocardial injury (infarction or surgical scar). The necrotic tissue is replaced by collagen fibrils that are cross-linked to provide mechanical stability in response to cardiomyocyte loss. On the other hand, *reactive* diffuse myocardial fibrosis (DMF) is characterized by a diffuse deposition of excess fibrous tissue relative to the mass of cardiomyocytes. In this case, MF appears as an accumulation of collagen fibers between individual cardiomyocytes and cardiomyocyte fascicles and around the intramyocardial vessels. As a result, the highly organized architecture of the myocardial interstitium is replaced with a thickened, poorly organized structure (11, 12) that alters the mechanical properties of the myocardium, thus impairing cardiac function and electrical activity, and contributing to the development of HF and the poor outcomes that characterize this condition.

Both types of MF can concur in the same individual, as seen in patients with ischemic cardiomyopathy that exhibit FMF at the necrotic site and DMF in the distant myocardium of both ventricles (13). Indeed, DMF can be facilitated by systemic factors activated secondarily to the loss of cardiac function (such as neurohormonal activation) (14) or related to extracardiac comorbidities (such as inflammation associated with metabolic syndrome or chronic kidney disease (15).

Beyond the quantity of fibrous tissue, the composition and physicochemical properties of the fibers are important in DMF. As previously mentioned, type I collagen fibers exhibit the highest stiffness, therefore, the ratio between type I and type III collagen will have an impact on overall myocardial stiffness. On the other hand, the type of intermolecular covalent bonding among collagen fibrils mediated by enzymes such as lysyl oxidases (LOXs), the so-called collagen cross-linking, will influence the resistance to degradation by matrix metalloproteinases (MMPs) and will also have an impact on myocardial stiffness.

Finally, it is well known that, coming from different embryonic origins, the left ventricle (LV) and the right ventricle (RV) respond distinctly to normal and abnormal loading conditions (16). Therefore, differences in the pattern and type of MF developed by both ventricles are also expected. The collagen content of a healthy RV is higher than that of the LV (17) and the RV and LV have different matrix metalloproteinase and ECM protein-expression patterns (18). Under the same overloading conditions, the RV exhibits less ability to develop adaptative remodeling (19, 20) leading to dilatation and dysfunction. This is particularly relevant in CHD, where morphologically RVs are exposed to extreme overloading conditions such as sustaining the systemic circulation, sometimes as a single ventricle, severe pulmonary hypertension or chronic pulmonary regurgitation as in repaired tetralogy of Fallot.

## Role of myocardial fibrosis in the development of heart failure and arrhythmia

### Diastolic dysfunction

**Figure 1:**
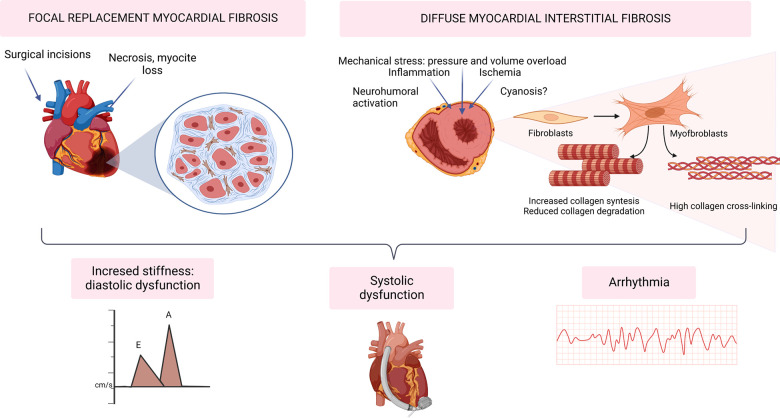
Myocardial fibrosis in the development of heart failure and arrhythmia in congenital heart disease. Created with BioRender.com.

Interstitial MF has classically been related to the development of diastolic impairment since an excessive accumulation of insoluble collagen in the ECM leads to an abnormally stiff myocardium (see Figure 1).

In a physiological state, collagen fibers form a deformable elastic structure that stores energy when deformed from its neutral position during systolic contraction. Lengthening of the myocardium during diastole is then facilitated by the release of potential energy stored during systolic compression of these fibers, generating a suction effect that allows early diastolic filling (21).

When extracellular viscoelastic properties are impaired this suction effect is compromised. This leads to a higher dependence on atrial contraction and elevated filling pressures to maintain the stroke volume, which can also cause venous congestion and ultimately result in congestive heart failure.

Aside from the absolute amount of collagen, myocardial stiffness also depends on the characteristics of the fibers in the extracellular matrix. Thus, a higher fiber linkage and a higher proportion of type I collagen have also been correlated with impaired diastolic parameters in echocardiography, elevated peptides or elevated filling pressures in right heart catheterization (22–27).

### Systolic dysfunction

In this setting, MF might just be part of the reparative response to the loss of contractile mass, becoming a marker of more advanced cardiomyopathy (28). However, the architecture of myocardial fibrillar collagen is also important in systolic performance, as it facilitates transduction of cardiomyocyte contraction (29, 30). Furthermore, the reduced stretching of the fibers in diastole can also compromise systolic function by altering the length-dependent cardiac muscle fiber shortening related to the Frank-Starling law.

### Arrhythmias

Both FMF and DMF impair myocardial electrophysiology *via* several mechanisms, constituting an important arrhythmic substrate. Beyond re-entry tachycardias around macroscopic scars (e.g., surgical incisions), DMF interferes with myocardial electrophysiology by slowing down action potential propagation, initiating re-entry, promoting after-depolarizations, and increasing ectopic automaticity (31, 32).

Interestingly, some studies have shown that tachyarrhythmias can initiate or exacerbate MF by activation of profibrotic and proinflammatory signaling. This mechanism would perpetuate electric disturbances while contributing to the impairment of ventricular function (33–35).

## Diagnosing myocardial fibrosis

Endomyocardial biopsy with histopathological analysis is the gold standard for MF diagnosis, but it presents several limitations as it is an invasive procedure and a difficult option in clinical practice (7, 36). Fortunately, currently available circulating and imaging biomarkers provide indirect MF diagnosis.

### Imaging tests

Cardiac magnetic resonance (CMR) methodologies for the detection of MF are based on the concept that fibrosis increases the extracellular space (37). As previously mentioned, MF can be divided into FMF, with dense macroscopic “replacement” fibrosis (scar) located to a specific, definable area; and DMF, which is more microscopic and uniform, with a global distribution and typically in response to chronic abnormal loading conditions (38–40).

There are two major CMR techniques used for MF assessment: late gadolinium enhancement (LGE) imaging and T1 mapping/extracellular volume (ECV) fraction calculation.

#### Late gadolinium enhancement

LGE is a CMR technique that identifies areas of discrete replacement fibrosis. Image acquisition is typically performed 10–20 min after administration of a gadolinium-based contrast agent. In damaged myocardial tissue, where there has been a significant localized expansion of the extracellular matrix, the distribution volume of the contrast agent is increased and the wash-out delayed (41).

LGE requires spatial heterogeneity to detect focal fibrosis, being a dichotomous method. It has the advantage that it analyzes the entire cardiac image, without focusing on a specific area, so it could be more sensitive for overall fibrosis burden, even though it requires a greater amount of fibrosis to be detectable visually (37).

#### Extracellular volume and T1 mapping

ECV mapping quantifies MF by measuring the extracellular compartment depicted by the myocardial uptake of contrast relative to plasma (42). ECV represents the ratio of the interstitium relative to the total myocardial mass.

ECV has advantages over LGE, being intrinsically quantitative and able to assess both focal and diffuse fibrosis. However, although fibrosis is certainly a cause of increased ECV, it is not the only one, as extracellular space expansion may be due to other causes (myocardial vasculature, nonfibrillar proteins, edema, amyloidosis, etc.,) (7, 42, 43).

Even so, ECV is the most widely used imaging biomarker for interstitial fibrosis (41) as it has been extensively validated against the collagen volume fraction (44, 45), it is highly reproducible across different CMR scans (46), it can predict outcomes (47, 48) and histologic validation data show overall the best agreement with ECV compared with other T1 based metrics (46, 49). While native (precontrast) T1 increases and postcontrast T1 decreases with MF, they are inferior MF measures compared with ECV (42, 50). Native T1 reflects changes involving the whole myocardium (intracellular and extracellular compartments) and several confounders affect postcontrast T1 (clearance, body weight, anemia …). Despite this, some studies found a good correlation between native and post-contrast T1 and myocardial fibrosis (51, 52).

Both ECV and T1 are quantitative measures that can allow us to grade disease activity, monitor progress and guide treatment (53). However, the measurements are sensitive to many scanner parameters, with no consensus regarding a vendor-neutral standard approach to image acquisition and postprocessing, so standardization against healthy volunteers is recommended for each CMR scanner and each specific protocol (51, 54). The wide variety of technical and methodological bias-causing confounders makes it difficult to both directly compare native T1 and ECV values between studies.

#### Characterization of fibrosis with CMR in CHD

MF is the final common pathway of a variety of congenital lesions. It is found in both the right and left ventricles and is not limited to areas of surgical scarring or within a coronary distribution (38) (FMF), but it also occurs more diffusely (DMF) in the setting of volume and/or pressure overload (49).

**Figure 2:**
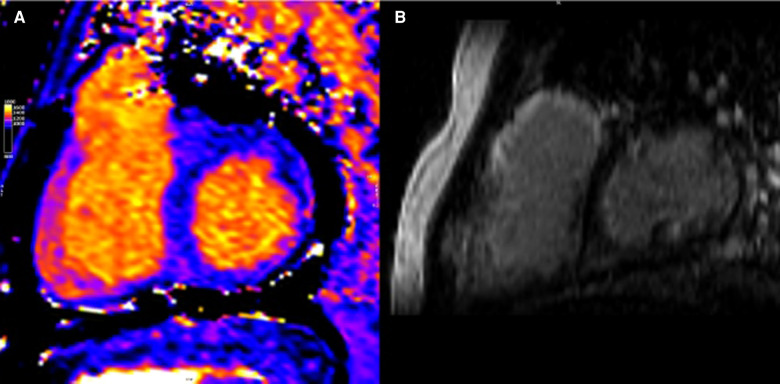
CMR of patient with repaired tetralogy of Fallot. (**A**) Precontrast T1 mapping of 1,091 ms (normal range of 950–1,050 ms) (**B**) Extent LGE, especially anterior wall of RVOT and anterior wall of RV.

Several studies have characterized MF by CMR in CHD (39, 55); however, there are several limitations. RV disease is hard to assess due to the small thickness of the RV wall, approaching the T1 mapping spatial resolution limit (49). Even in patients with RVs that support systemic circulation, the RV free wall has been deemed difficult to measure by T1 mapping (56) (see Figure 2). Some studies focus on a single mid-ventricular plane, which may not be representative of the entire ventricle, and many CHD patients have limitations for CMR scanning (device electrodes and young age or syndromic problems (38, 57).

CHD patients present certain peculiarities and common challenges to CMR when studying MF, such as wide and abnormally shaped QRS complexes, breath-holding difficulties, metallic artifacts from sternal wires and transcatheter devices, as well as pathology focused on the thin-walled RV (39).

### Circulating biomarkers

**Figure 3:**
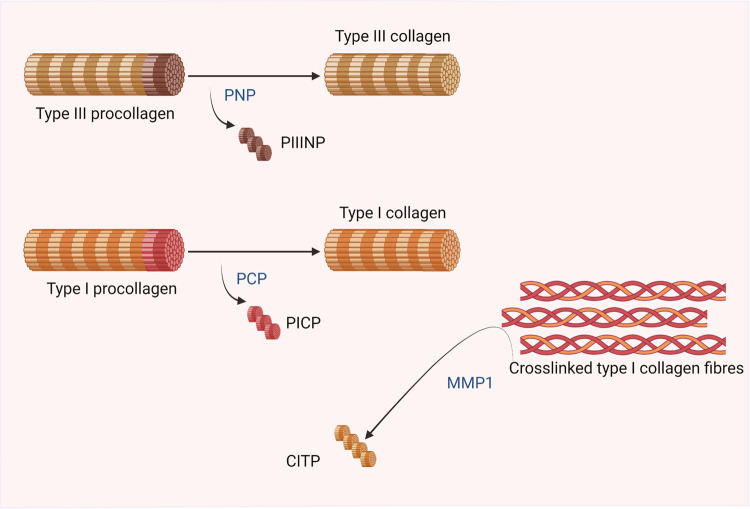
Biomarkers of collagen metabolism. Created with BioRender.com.

Multiple circulating biomarkers of collagen metabolism have been described, but only a few molecules have demonstrated an association between the biomarker levels and histologically assessed myocardial collagen deposition or collagen cross-linking (58–61) (see Figure 3). A positive gradient has been found from the concentration of some of these biomarkers in coronary sinus blood toward the concentration in peripheral vein blood in patients with different types of cardiac disease (58); however, none were cardiac-specific (7).

The biomarkers that have shown more consistent results and a higher pathological correlation with MF upon biopsy are the carboxy-terminal propeptide of type I procollagen (PICP), the amino-terminal propeptide of type III procollagen (PIIINP) and the ratio of serum carboxy-terminal telopeptide of type I collagen to serum matrix metalloproteinase-1 (CITP:MMP-1ratio) (7, 40). The first two are biomarkers of collagen synthesis that directly correlate with the deposition of collagen fibers in the myocardium in different heart diseases. The CITP:MMP-1 ratio inversely correlates with myocardial collagen cross-linking (59, 62). Therefore, low levels of the CITP:MMP-1 ratio and high levels of PICP and PIIINP would indicate DMF.

#### Carboxy-terminal propeptide of type I procollagen

PICP is generated during the extracellular maturation of the collagen molecule by conversion of procollagen type I into collagen type I by the enzyme bone morphogenetic protein-1 or procollagen carboxy-terminal proteinase (63).

Serum levels of PICP have been associated with various findings: myocardial deposition of collagen type I fibers in HF patients (23); correlation with severity of HFrEF; with mortality in HFpEF and HFrEF (64, 65) and changes after treatment with torasemide and spironolactone in heart failure (66, 67).

#### Amino-terminal propeptide of procollagen type III

Most serum PIIINP is generated during the extracellular conversion of procollagen type III to collagen type III by the enzyme procollagen amino-terminal proteinase (63). An association has been found between serum PIIINP and myocardial deposition of collagen type III fibers in HF patients and with severity and outcomes in HF of different causes, regardless of the ejection fraction (64, 68).

#### Carboxy-terminal telopeptide of type I collagen to matrix metalloproteinase-1 ratio

Once the collagen molecule is mature, the spontaneous self-assembling of molecules results in the formation of collagen fibrils. In a second step, the formation of intra- and intermolecular covalent bonds, cross-links, between lysine residues ensues.

Collagen cross-linking determines the resistance of collagen fibers to MMP degradation; therefore, the highly cross-linked type I collagen fibers have increased resistance to degradation by MMP1, producing a reduction in collagen type I telopeptide (CITP) levels and, thereby, a decreased CITP:MMP1 ratio. Thus, the serum CITP:MMP-1 ratio inversely correlates with myocardial collagen cross-linking (59).

A low CITP:MMP-1 ratio has been associated with a higher risk of hospitalization for HF and cardiovascular mortality in hypertensive patients (59, 69). It also identifies HFpEF patients resistant to the beneficial effects of spironolactone (70).

## Relevance of myocardial fibrosis in congenital heart disease

Myocardial fibrosis is the final common pathway of a variety of CHDs due to the multiple procedures and situations of volume and pressure overload to which these hearts are subjected throughout their lives. The current evidence regarding myocardial fibrosis in specific congenital heart conditions will be reviewed in this chapter, focusing on the most common congenital cardiac disorders.

### Tetralogy of Fallot

**Figure 4:**
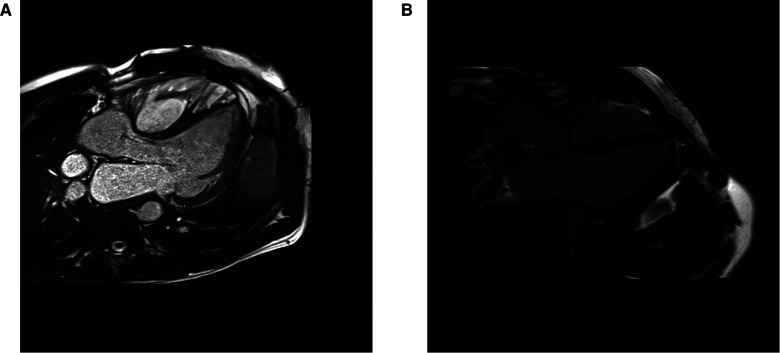
CMR cine 3 chamber view (**A**) and LGE image (**B**) from a 39 years old patient with a repaired tetralogy of Fallot, showing focal fibrosis in the anterior wall of the right ventricle.

There is histological evidence of increased interstitial collagen deposition in patients with tetralogy of Fallot (TOF). Existing data suggest that chronic exposure to cyanosis and pressure overload play a role in the development of MF before repair (71). Unloading the RV with a complete repair seems to have a beneficial effect; necropsy studies show a greater extent of MF in unrepaired than repaired TOF (72). However, even after reparative surgery, MF seems to progress in both ventricles and is associated with worse outcomes (72, 73), probably due to residual overloading conditions, usually severe pulmonary regurgitation (see Figure 4).

In 2006, Babu-Narayan et al. (74), published the first study to detect FMF in repaired TOF using LGE. FMF was not only found in areas with patch repair but also distant areas of the RV and even the LV. The extent of LGE was related to age, functional class, neurohormonal activation, ventricular dysfunction and clinical events, including exercise intolerance and clinical arrhythmias. Several studies have since followed, collectively confirming that a greater degree of fibrosis is associated with adverse clinical outcomes (80–82). Additional studies have shown that the presence of fibrosis is not limited to the RV but is also seen in the LV (37), with a positive correlation between LV and RV ECV values, thus indicating various coupling mechanisms and an adverse ventricular-ventricular interaction at the tissue level (73, 75).

RV LGE has been related to RV diastolic dysfunction (83) and fragmentation of the QRS complex, a marker of ventricular arrhythmias in this patient population (84). In a recent study (85), the extent of RV LGE and presence of LV LGE were included in a score that integrated multiple, appropriately weighted, risk factors and which identified the subgroup of patients at the highest annual risk of death.

DMF has also been studied in patients with TOF. Chen and colleagues (73) found a correlation between RV and LV ECVs and an association between predominant volume overloading hemodynamic burden and RV ECV, in contrast with previous evidence supporting pressure overload as the main driver of MF. Since ECV represents the ratio of interstitium relative to total myocardial mass, the authors attributed this finding to a potential myocyte loss as a maladaptative mechanism of cellular remodeling in repaired TOF patients with chronic pulmonary regurgitation. Subsequent studies have also found prolonged RV native T1 times in repaired TOF with volume overload, both in pediatric (86) and adult cohorts (77), which would indicate the presence of DMF. Interestingly, a shorter native T1 was observed in patients with pulmonary valve replacement. This finding, together with histological evidence that patients with repaired TOF and residual pulmonary regurgitation, but reasonably preserved RV volumes and function, show no significant differences in collagen content and LOX activity when compared to healthy controls (87) suggests that DMF might be reversible and even have an oscillating behaviour according to the adaptative capacity of the myocardium to successive overloading conditions.

Elevated RV and LV ECVs have been associated with older age, age at TOF repair, neurohormonal activation, longer QRS duration, shorter six-minute walk distance, larger left atrial volume and adverse outcomes, including death, congestive heart failure and arrhythmias (73, 75, 76).

There are some studies with negative results. Using native T1 mapping, Yigit et al. (88), found focal fibrosis at the RV insertion points and the LV walls in a population of 35 patients with repaired TOF. However, it was not possible to detect RV outflow tract fibrosis with this technique, possibly due to the aforementioned technical limitations of the RV (88).

As for circulating biomarkers, patients with repaired TOF exhibit a pattern of excessive collagen synthesis and dysregulated degradation, with elevated circulating PICP and PIIINP levels and PICP:CITP ratio, a positive correlation between collagen synthesis biomarkers (PICP and PIIINP) and RV end-diastolic volumes (EDV) and a negative correlation with RV systolic function (78, 79).

### Systemic right ventricle

Atrial switch operation in patients with D-transposition leaves the RV as the systemic pump (89, 90), as it happens naturally in patients born with congenitally corrected transposition of the great arteries (ccTGA) In both situations, a progressive systemic right ventricle (sRV) dilatation and dysfunction are the norm, leading to reduced exercise capacity, HF and arrhythmia (91–93) (see table 2).

**Table 1:** Most relevant studies characterizing myocardial interstitial fibrosis with cardiac magnetic resonance and collagen turnover biomarkers in patients with repaired tetralogy of Fallot.

	Reference	Year	Population	Characterization of fibrosis	Key clinical findings
CMR with LGE	Babu-Narayan, et al. (74)	2006	*N* = 92 32.2 ± 11 years	RV LGE was present in all patients at surgical sites, and common in the inferior RV insertion point (79%) and trabeculated myocardium (24%). LV LGE (53%) was located at site of the apical vent insertion (49%), in the inferior or lateral wall consistent with infarction (5%), or in other areas (8%)	Patients with supramedian RV LGE score were older and more symptomatic. A greater extent of RV LGE correlated with higher levels of BNP, exercise intolerance, RV dysfunction and clinical arrhythmia. Non–apical vent LV LGE also correlated with markers of adverse outcome.
CMR with ECV	Chen, et al. (73)	2016	*N* = 84 23.3 years (16.3–32.8)	LV ECV above the upper limit of normal was observed in 11 patients and for RV ECV in 9 patients	Greater RV ECV was associated with having volume overload as the predominant hemodynamic burden (in contraposition to pressure-overload or mixed lesions) Increased LV ECV was independently associated with arrhythmia,
	Broberg, et al. (75)	2016	*N* = 52 40 ± 14 years.	LV ECV was greater in TOF than in control subjects	LV ECV associated with adverse clinical markers and outcomes (arrhythmia, cardiovascular death)
	Hanneman, et al. (76)	2018	*N* = 44. 32.9 ± 13.6 years.	RV ECV was higher in patients compared with the controls.	RVECV correlated with adverse events (death, cardiac arrest, heart failure and ventricular tachycardia). LVECV was not associated with adverse events
	Cochet, et al. (77)	2019	*N* = 103. 28 ± 15 years	Higher T1 and ECV in both ventricles.	LV native T1 was independent correlate of ventricular arrhythmia Patients with a history of pulmonary valve replacement showed larger scars on RV out-flow tract but shorter LV and RV native T1.
Collagen turnover biomarkers	Lai, et al. (78)	2011	*N* = 39 17.7 ± 4.1 years	High PICP and PIIINP levels	PICP and PIIINP levels correlated with age, body mass index and parameters of RV and LV systolic function.
	Chen, et al. (79)	2013	*N* = 70	Higher PICP levels, PICP:CITP ratios and TIMP-1 concentrations	PICP levels correlated with higher RV LGE scores, lower VO2max and larger RV volumes.

**Table 2:** Most relevant studies characterizing myocardial interstitial fibrosis with cardiac magnetic resonance and collagen turnover biomarkers in patients with systemic right ventricle.

	Reference	Year	Population	Characterization of fibrosis	Key clinical findings.
CMR studies with LGE	Babu-Narayan et al. (94)	2005	36 d-TGA patients. 27 ± 7 years old.	61% of patients showed LGE in the sRV.	Patients with LGE were older, had increased RVESV, decreased SRV EF, increased QRS duration and increased QT dispersion. Arrhythmias were more prevalent in the LGE group.
	Giardini et al. (95)	2006	34 patients with sRV (23 d-TGA; 11 cc-TGA;) 25 ± 8 years old	41% of patients showed LGE in the sRV.	LGE was associated with older age, a lower sRV EF, higher RV wall stress, reduced peak oxygen uptake and history of arrhythmia
	Fratz et al. (96)	2006	27 sRV patients (18 atrial switch, 9 ccTGA) 23.4 ± 5.3 years old.	Only one subendocardial scar in a patient with cc-TGA.	
	Rydman et al. (97)	2015	55 patients 27 ± 7 years old.	RV LGE was present in 56% of patients..	Followed for a median 7.8 years; LGE was strongly associated with adverse clinical outcome, mainly atrial arrhythmia
	Babu-Narayan et al. (98)	2016	22 d-TGA patients 28 ± 8 years of age	LGE was found in 59% of the patients.	LGE was related to sRV dyssynchrony, reduced systolic function, lower exercise capacity, more severe tricuspid regurgitation and more previous arrhythmia
	Ladouceur et al. (99)	2018	48 patients with d-TGA. 32 ± 3 years old.	LGE was found in 35% of patients	Correlated with sRV wall stress
CMR studies with ECV	Plymen et al. (56)	2013	14 D-TGA patients. 33.7 + 6.5 years old.	Higher ECV at the mean septal level in comparison with healthy controls. This sRV population did not have areas of LGE and had normal EDV and systolic function.	ECV correlated with NT-proBNP levels
	Broberg et al. (100)	2018	53 subjects with sRV (43 d-TGA, 10 ccTGA). Age 34.6 ± 10.3 years,	28% had an elevated ECV for the sRV.	Those with an elevated ECV had higher BNP levels. After a median follow up of 4.2 ± 1.9 years those with elevated ECV presented more cardiovascular events (new arrhythmia, arrhythmia device, HF hospitalisation, listing for transplantation, mechanical support or cardiovascular death)
	Shehu et al. (101)	2018	10 d-TGA patients. 36.8 ± 5.3 years old	ECV of the inferior wall of the LV was significantly increased compared to the ECV of the sRV.	
Collagen turnover biomarkers	Dos et al. (102)	2013	26 patients with atrial switch, randomised to eplerenone or placebo. 26.4 ± 6.4 years old.	The whole cohort showed higher levels of CICP, ICTP than healthy controls. Galectin 3 and TIMP1 lower than controls.	In patients under eplerenone, a trend toward a reduction in CICP, NTproMMP1,TIMP1 and galectin 3 levels and a lower increase in ICTP
	Lipczynska et al. (103)	2017	56 patients with D-TGA 25.6 ± 4.8 years old.	Finding elevated levels of several biomarkers (TIMP1,PIIINP,CITP,PINP) when compared with healthy controls	PIIINP: good marker of sRV remodelling. MMP-9and TIMP-1 predicted adverse clinical outcomes.
	Ladouceur et al. (99)	2018	48 patients with D-TGA	Increased MMP1/TIMP1 ratio	MMP1/TIMP1 correlated with higher sRV wall stress and lower sRV EF

The first cross-sectional studies characterizing sRV fibrosis *via* MRI (94–96, 98, 99) showed that the presence of late gadolinium enhancement is common in sRV, ranging from 5% to 60% of patients, probably reflecting methodological differences and heterogenic cohorts. These studies correlated the presence and extent of LGE with functional parameters such as higher sRV end-systolic volume index and lower sRV ejection fraction (EF). Furthermore, the patients more commonly presenting LGE had a prior history of arrhythmia and a lower exercise capacity.

Subsequently, the presence of LGE was validated as a prognostic marker in longitudinal studies. LGE was found to be a good predictor of new onset arrhythmia and heart failure in this population (97).

Lipczynska and colleagues (103) proved that collagen turnover biomarkers were also valuable as prognostic markers in patients with sRV. PIIINP was postulated as a good marker of sRV remodeling, as it correlated with higher sRV mass, higher sRV EDV and worse global longitudinal strain. Furthermore, MMP-9 and tissue inhibitor of metalloproteinase 1(TIMP-1) predicted adverse clinical outcomes in this cohort.

Different mechanisms are postulated to explain late sRV failure and the presence of scarring, which are probably multifactorial. Preoperative hypoxemia and deficient myocardial protection in older cohorts may play a role since more extensive fibrosis has been found in older patients and patients with a late repair (94, 95, 98, 99). A deficient coronary supply for a thickened sRV may be another explanation. Ladouceur and co-workers (99) found that sRV fibrosis was related to increased RV wall stress. Initially, the increased pressure overload for a morphological RV in the systemic position would be compensated by an adaptive hypertrophy to preserve RV function and normalize RV wall stress. However, with time, sRV maladaptation would lead to excessive hypertrophy, which would present demand-supply mismatch ischemia, resulting in the presence of patchy fibrosis and ultimately sRV systolic and diastolic dysfunction. Some studies correlated LGE with QRS duration and QT dispersion (94, 95) and, in the study by Babu-Narayan and colleagues, LGE correlated with echocardiographic parameters of electromechanical delay and dyssynchrony (98), this being another possible mechanism for the deterioration of myocardial function and arrhythmia development.

Recently, CMR studies with T1 mapping techniques for DMF characterization yielded interesting results. Plymen and colleagues (56) found a higher ECV at the mean septal level in a small cohort of 14 patients with sRV in comparison with healthy controls. Interestingly enough, this sRV population did not have areas of LGE and had normal EDV and systolic function (mean RVEDV 79 ml/m^2^ and mean sRV EF 59%). This could imply that DMF is present in the early stages of the disease and even in the absence of macroscopic scars.

Broberg and co-workers (100) studied 53 subjects with sRV (43 D-TGA, 10 ccTGA);of these, 28% had an elevated ECV value based on gender-specific cut-offs for the systemic LV of healthy controls. No differences were found in sRV volume or EF, but patients with elevated ECV had higher levels of serum brain natriuretic peptide (BNP) and presented more cardiovascular events (new arrhythmia, arrhythmia device, HF hospitalization, listing for transplantation, mechanical support or cardiovascular death)after a median follow up of 4.2 ± 1.9 years. Events in those with elevated ECV tended to occur early and be more related to HF, whereas events in the normal ECV group occurred later and were more often atrial arrhythmias.

More intriguing results focusing on the subpulmonic LV were published recently (101, 104). As in previous studies, a greater native T1 and ECV were found in the sRV, but the authors also found elevated ECV in the subpulmonic LV compared with healthy controls. Moreover, a greater segment and average ECV from the LV was found than in the sRV. The cause of these findings remains poorly understood, although there are several theories, including chronic volume unloading of the eccentrically compressed left ventricle, prevalent postcapillary pulmonary hypertension or ventricular-ventricular interaction at the extracellular matrix level. It also remains unclear whether fibrosis in the subpulmonic LV may play a role in prognosis, but it warrants further research as adverse remodeling with an overt increase in systolic and diastolic volumes of the subpulmonic LV is associated with a worse clinical outcome (105, 106).

### Arterial switch operation

The arterial switch operation (ASO) is the current procedure of choice for patients born with D-TGA. In this population, the systemic LV is usually believed to be normal (107–110). However, several factors might impair its myocardial function in the long-term, such as neonatal cyanosis, neonatal cardiopulmonary bypass, impaired coronary perfusion, myocardial denervation, aortic regurgitation or increased aortic stiffness (111) (see Table 3).

**Table 3:** Most relevant studies characterizing myocardial fibrosis in patients with arterial switch operation.

	Reference	Year	Population	Characterization of fibrosis	Key clinical findings
CMR with LGE	Shepard, et al. (115)	2016	220 patients (mean age of 15.4 years)	Myocardial scarring characterized by LGE was found in only 1.8% of patients.	LGE was not common even when 26% of patients had some degree of left ventricular dilatation and 21.5% of patients had some degree of left ventricular dysfunction.
CMR with ECV	Grotenhuis, et al. (116)	2019	30 patients (mean age 15.4 years)	No LGE was found. LV native T1 times were prolonged in ASO patients and correlated with the LV mass/volume ratio. No differences were found in ECV.	Neither ECV nor T1 times correlated with LV function.
	Suther, et al. (117)	2019	30 patients (mean age 11.7 years)	No LGE was found. Slight increase in ECV compared with healthy controls in all coronary territories	

Indeed, in the last few years, data with speckle tracking echocardiography have emerged suggesting that a mild impairment of LV function may be more common than previously thought (112–114).

A single-center CMR cohort study (115) in 220 patients (mean age of 15.4 years) found that 26% of patients had some degree of left ventricular dilatation, while 20% had right ventricular dilatation. Left ventricular dysfunction was present in 21.5% of patients (mild in most cases), and only 5.1% of patients had mild right ventricular dysfunction; however, myocardial scarring characterized by LGE was found in only 1.8% of patients.

Diastolic function may also be impaired. Some studies found slight differences in terms of ventricular stiffness and diastolic parameters when compared with healthy controls, but still within the normal range, and in the absence of an established LV hypertrophy (118). This could be explained by an intrinsic stiff myocardium with some degree of fibrotic remodeling, suggested by increased fibrosis markers in CMR studies. Grotenhuis and colleagues (116) studied a cohort of patients without myocardial scarring or perfusion defects and with a normal LV ejection fraction. However, compared with healthy controls, end-diastolic and end-systolic volumes were increased and an altered contraction pattern was observed, the longitudinal strain being lower while the circumferential strain was higher at all short-axis levels, maybe reflecting subclinical compensated systolic dysfunction. LV native T1 times were prolonged in ASO patients and correlated with the LV mass/volume ratio, suggesting the presence of a certain degree of myocardial fibrosis. However, no differences were found in ECV, and neither ECV nor T1 times correlated with LV function. On the other hand, Suther and co-workers detected a slight increase in ECV in a small cohort of adolescents with ASO and normal coronary arteries compared with healthy controls (117). Whether these findings precede the development of overt LV dysfunction over time remains speculative and multicentric longitudinal studies will be required to clarify their prevalence and their prognostic significance.

### Chronic cyanosis

**Figure 5:**
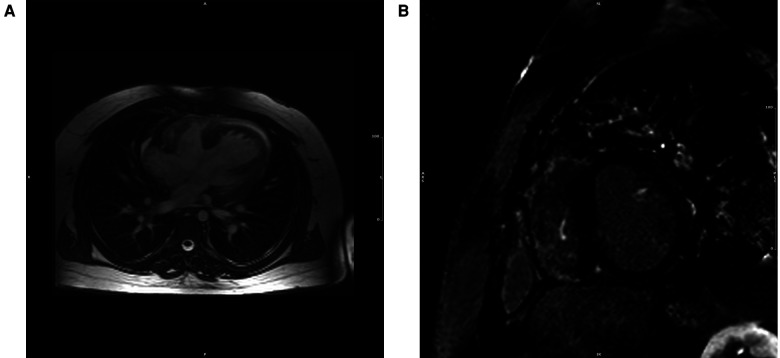
CMR cine (**A**) and LGE (**B**) image from 27-year-old patient Eisenmenger syndrome because of an untreated ventricular septal defect, showing extent in right ventricular free wall, as well as in the insertion points and in the left ventricle's papillary muscles. The patient developed ventricular arrhythmias in the follow-up and died suddenly at the age of 33.

The assumption that cyanosis causes diffuse intramyocardial fibrosis has been accepted for decades, justified by the findings of fibrosis in specimens from autopsies of patients with unrepaired cyanotic heart disease (see Figure 5 and Table 1). Nevertheless, it remains unclear whether these findings could be related to the presence of adverse hemodynamic loading conditions, the cyanotic condition itself *via* activation of hypoxia-inducible factors (119, 120) or both.

In the last few years, this hypothesis has been challenged by some MRI studies mainly focused on patients with Eisenmenger syndrome. However, data are scarce and there are some discrepant results (see Table 4).

**Table 4:** Most relevant studies characterizing myocardial fibrosis in patients with chronic cyanosis, Eisenmenger syndrome and Fontan circulation.

	Reference	Year	Population	Characterization of fibrosis	Key clinical findings
CMR with LGE	Rathod, et al. (121)	2010	*N* = 90 Fontan patients 23.1 ± 10.9 years	LGE was present in 28% of this population, the vast majority in the free wall of the predominant ventricle	LGE presence correlated with lower ejection fraction, increased ventricular volumes and mass. Patients showing LGE had more non-sustained ventricular tachycardia (36% vs. 11%; *p* = 0.01).
	Broberg, et al. (122)	2014	*N* = 45 Eisenmenger syndrome patients. Age: 43 ± 13 years	73% showed LGE in the right ventricular myocardium (70%) but also in the left ventricle (33%)	LGE could not be correlated with age, arrhythmia, oxygen saturation, haemoglobin levels, ventricular performance or exercise capacity.
CMR with ECV	Broberg, et al. (55)	2010	*N* = 50 CHD patients (10 cyanotic) Age: 37 ± 12 years	Among the entire cohort, ECV values were higher in patients with systemic RV and cyanotic patients	There was a strong correlation between ECV and systemic indexed EDV and EF. No correlation between the fibrosis index and resting oxygen saturation or age.
	Kato, et al. (123)	2017	*N* = 21 Fontan patients. Age: 9.7 ± 4.6 years	Higher ECV and prolonged native T1 times in patients with a morphologically right single ventricle compared to those with a morphologically left single ventricle.	Age at bidirectional cavopulmonary connection was correlated with T1
	Pisesky et al. (124).	2021	*N* = 55 Fontan patients Mean age: 14 years	Higher ECV and prolonged native T1.	Correlation between native T1 and the composite end point of cardiac readmission, cardiac reintervention, Fontan failure or any clinically significant arrhythmia

Broberg and collaborators (55, 122) quantified ECV in a cohort of 50 CHD patients, 10 of which were cyanotic. Among the entire CHD cohort, cyanotic patients was the group with the second highest ECV, after patients with systemic right ventricle. There was a strong correlation between ECV and systemic indexed EDV and EF. In a larger study from the same group (122) in 45 subjects with Eisenmenger syndrome, LGE was present in 22/30 (73%) patients, specially in the right but also in the left ventricular myocardium. In none of those works fibrosis could be correlated with age, history of arrhythmia, oxygen saturation, hemoglobin levels, exercise capacity or mortality.

On the other hand, Kharabish et al. (125), performed contrast CMR in a very small cohort of patients with repaired and unrepaired cyanotic heart disease. Surprisingly, untreated patients had a significantly lower left ventricular ECV percentage than the repaired patients, and no significant differences were found between cyanotic patients and normal controls.

Both studies were limited by the small cohort size and the heterogeneity of the underlying anatomy, age and physiological state of the patients. Larger multicenter studies are required to unravel the relevance of MF in the pathophysiology of chronic cyanosis and its role in sudden cardiac death and HF, the two leading causes of mortality in this patient population (126).

### Fontan circulation

Myocardial fibrosis has been less studied in this patient population but new techniques for detecting diffuse fibrosis have increased the interest in univentricular patients.

Rathod and colleagues (121) detected FMF in 28% of a population of 90 patients with Fontan circulation. As expected, FMF was found at the surgical sites but the vast majority of LGE distribution was found in the free wall of the predominant ventricle. LGE presence correlated with adverse ventricular mechanics and was associated with non-sustained ventricular tachycardia. Likewise, LGE has been associated with time to death or transplant in this patient population (127).

DMF has also been evaluated in Fontan patients; compared with healthy controls, they presented higher ECV and native T1. Additionally, there was a correlation between native T1 and aortopulmonary collateral flow and an association with the composite end point of cardiac readmission, cardiac reintervention, Fontan failure or any clinically significant arrhythmia (124). However, Kato and colleagues could only find higher ECV in Fontan patients with morphologically right single ventricles. In these cohort, no difference was found regarding T1 or ECV between healthy controls and Fontan patients with morphologically left single ventricles (123).

A recent study using circulating biomarkers in 25 Fontan patients observed a significant correlation between ECV and systemic ventricular end-diastolic pressure and between ECV and liver stiffness. Furthermore, patients with elevated ECV had elevated MMPs and TIMPs, and these patients presented greater liver stiffness (128).

Whether all these findings are related to the intrinsic peculiarities of Fontan circulation, the previous cyanotic phase or both remains to be determined.

## Therapeutic options and future directions

As previously mentioned, fibrosis is a highly dynamic process that typically involves the recruitment of fibroblasts and their conversion into myofibroblasts, excessive ECM synthesis and secretion, ECM protein cross-linking, dysregulation of ECM production and breakdown by MMPs and their endogenous inhibitors. Therefore, multiple targets or therapeutic opportunities may exist. In addition, different types of fibrosis (FMF vs. DMF) and different stages of the fibrotic process (more static vs. more dynamic) may coexist in the same individual and identification of the specific or dominant type of fibrosis would be important for the selection of the anti-fibrotic approach. Timely detection of fibrosis and determination of its state could potentially help diagnose and halt HF progression.

### Targeting the fibrotic process

There are several potential novel therapeutic strategies for MF coming from the idiopathic pulmonary fibrosis armamentarium (Transforming growth factor beta inhibitor pirfenidone; tyrosine kinase inhibitor nintedanib; pamrevlumab, a human monoclonal antibody that inhibits connective tissue growth factoractivity; simtuzumab, a humanized monoclonal antibody to block lysyl oxidase like 2), some of them showing promising results in preliminary studies (7). However, we still have limited knowledge of the diverse and heterogeneous mechanisms involved in the pathophysiology of the complex process of fibrosis and the different factors (setting, timing or etiology) that might jeopardize the final effectiveness of a given treatment. Also, the therapeutical target should be the excessive fibrous tissue without affecting the physiological collagen scaffold (12); the complexity of this is illustrated by a murine model of myocardial infarction developed by Clarke and colleagues (129). In this experimental model, MMP inhibition proved to be less effective than initially presumed. The authors postulated that early during the remodeling phase, MMP inhibition might be less effective because, although MMP levels are at their highest, there is scant collagen to degrade, while at later fibrotic phases it is less effective because, although collagen concentrations are high, MMP levels have fallen to low levels. On the other hand, MMPs have a full range of functions and myriad targets. All these factors may convey unpredictable and counterintuitive results when targeting the fibrotic process once it has been initiated.

### Targeting the triggers

While current experimental studies and even clinical trials [pirfenidone is currently being tested in the PIROUETTE (130) phase II trial] are underway to unravel these riddles, targeting the stimulus that triggered the maladaptative fibrotic process seems to be effective. There is evidence that some HF therapies (targeting neurohormonal activation) have antifibrotic effects (26, 67, 131, 132). However, in CHD, where clinical trials for HF therapy are extremely scarce, those focusing on MF as an endpoint are anecdotal. Our group developed a clinical trial evaluating the effect of the mineralocorticoid-receptor antagonist eplerenone on MF in patients with systemic RV (transposition of the great arteries repaired with the atrial switch procedure) (102). The study found an increased collagen turnover in this patient population compared with age- and gender-matched controls but was underpowered to show a reduction in ventricular mass after one year of treatment. The interpretation of the trend toward a reduction in CICP, MMP1, TIMP1 and galectin 3 levels observed in patients under eplerenone remains elusive as no T1 mapping was available at the time of the study to assess changes in DMF.

While the prognostic role of preoperative myocardial fibrosis in patient outcomes after valvular replacement has been extensively studied (133, 134), there is little information regarding MF reversibility after valvular replacement. However, recent data support the hypothesis DMF may reverse after unloading the ventricles both under volume and pressure overloading conditions (135, 136). The limited data on this topic focuses mainly on mitral and aortic valvular disease with no information, to the best of our knowledge, on right-sided valves. However, experimental studies (artery banding mouse models) suggest that reversibility of RV fibrosis after cessation of the overloading stimulus can also be achieved (137). On the other hand, preliminary and indirect data suggest that DMF in TOF might be reversible and even have an oscillating behavior according to the adaptative capacity of the myocardium to successive overloading conditions (77, 87).

### Differences between right and left ventricles

The RV and LV have distinct fibrotic responses and, therefore, may require different therapeutical approaches (138). Pirfenidone does not reverse RV fibrosis or enhance RV function in pulmonary artery-banded rats (139), but it does reduce RV fibrosis and remodeling in a murine model of non-severe pulmonary arterial hypertension (PAH) (140). In turn, eplerenone does not reverse RV fibrosis in Sugen-hypoxia and pulmonary artery-banded mice after PAH was established (141). On the other hand, whereas the microRNA miR-21, a known profibrotic agent currently studied as a target for the treatment of Alport Syndrome, seems to be crucially involved in the fibrotic response to mechanical and hormonal stimuli in isolated RV fibroblasts from dogs, this is not the case for dog LV fibroblasts (19). Whether these discrepant findings are related to the intrinsic different responses of both ventricles to overloading conditions or other determinants of the fibrotic process, such as timing or etiology, remain to be determined.

### Additional diagnostic methods

Wider availability of diagnostic methods to evaluate MF is needed. In recent years, cardiac computed tomography angiography (CTA) has yielded good results for MF assessment. An excellent correlation between ECV evaluated by CTA and by CMR has been shown in animal models, healthy volunteers and HF patients (142, 143). CTA-based quantification of ECV has also been validated against a histological assessment of myocardial fibrosis (142) and presented an association with adverse clinical outcomes after percutaneous aortic valve implant (144, 145). This technique may be of particular interest in patients with CHD since it overcomes some important caveats of CMR in this population: the shorter acquisition times make it easier for patients with cognitive impairment and patients with claustrophobia and it can be used in patients with paramagnetic metal implants.

On the other hand, new targeted approaches that have shown promising results for collagen detection in other organs, such as the novel molecular magnetic resonance contrast agents utilized in liver studies, might be applicable in heart diseases (146).

In summary, understanding HF pathophysiology lies beyond the mere assessment of cardiac function. In-depth consideration of myocardial structure and function at the tissue level, particularly of the myocardial interstitium, provides additional and crucial insight and may drive the development of new therapies. MF, particularly diffuse interstitial fibrosis, constitutes an emerging and promising therapeutic target in HF secondary to acquired heart conditions. Due to intrinsic particularities (preeminent involvement of overloaded morphologically RV, chronic cyanosis, etc.,), MF seems to play a determinant role in the development of HF and arrhythmias in CHD. Further studies in the field are needed to shed light on this exciting and promising area of research in CHD.
